# Metal Concentration Assessment in the Urine of Cigarette Smokers Who Switched to Electronic Cigarettes: A Pilot Study

**DOI:** 10.3390/ijerph17061877

**Published:** 2020-03-13

**Authors:** Adam Prokopowicz, Andrzej Sobczak, Jerzy Szdzuj, Katarzyna Grygoyć, Leon Kośmider

**Affiliations:** 1 Institute for Ecology of Industrial Areas, 6, Kossutha St., 40-844 Katowice, Poland; 2 Department of General and Analytical Chemistry, Faculty of Pharmaceutical Science in Sosnowiec, Medical University of Silesia, Jagiellonska 4 St., 41-200 Sosnowiec, Poland; andsobcz@poczta.onet.pl (A.S.); lkosmider@sum.edu.pl (L.K.); 3 Institute of Environmental Engineering, Polish Academy of Sciences, M. Skłodowskiej-Curie 34 Str., 41-819 Zabrze, Poland; jerzy.szdzuj@ipis.zabrze.pl (J.S.); katarzyna.grygoyc@ipis.zabrze.pl (K.G.)

**Keywords:** electronic cigarettes, metals, elements, urine

## Abstract

*Background*: E-cigarettes (ECs) seem to be a less harmful alternative for conventional cigarettes, however, very little is still known about the exposure to some elements, which are the components of this device and may contaminate the nicotine liquid. The aim of this study is to assess whether e-cigarette users are more exposed to 12 elements detected in aerosol than non-smokers and conventional cigarette smokers, using their concentrations in urine as exposure biomarkers. *Methods:* A cross-sectional, group-based survey was carried out using 90 volunteers classified into groups of non-smokers, EC-only users, dual EC users-cigarette smokers and cigarette-only smokers. Using inductively coupled plasma mass spectrometry (ICP-MS) and electrothermal atomic absorption spectrometry (ETAAS), Cr, Ni, Co, Ag, In, Mn, Ba, Sr, V, Sb, Cd and Pb levels were measured in spot urine samples. Among the selected groups, a comparison was made using the analysis of covariance and correlations with EC usage pattern were assessed by multiple linear regression. *Results:* Element concentrations in urine of EC-users were not significantly different from the levels found in non-smokers and smokers. Only in the case of Ba, Ni and Sb was a significant correlation found in relation to some e-cigarette usage patterns. *Conclusion:* Transfer of the investigated elements to the EC aerosol was not found to be a substantial source of exposure in EC users who quitted smoking.

## 1. Introduction

Electronic cigarettes (ECs), which have been introduced on the market for at least a decade, are considered a less harmful alternative to conventional cigarettes [1,2]. Some studies, however, demonstrate the possibility of higher exposure to some elements, which are the components of an EC device and contaminate nicotine liquids. Williams et al. [3] found that the concentrations of nine of eleven elements in the EC aerosol were higher than or equal to the corresponding concentrations in conventional cigarette smoke. In their latest investigations, aerosols from disposable electronic cigarettes/electronic hookahs were even more abundant in the total number of different elements than conventional tobacco smoke and some of them were found to be present in significantly higher concentrations [4]. Elements such as Cd, Pb, Ni, Sn, Cu, Cr, Sb, Mn, and Al were detected in both e-liquids and generated aerosols [3,4,5,6,7,8]. A dramatic increase was observed by Olmedo et al. in tank samples for Cr, Cu, Ni, Pb and Zn as well as in aerosol samples for Pb, Zn, Cr, Ni, and Sn. Furthermore, for such metals as Mn, Al, Cd, Sb, the concentrations were higher in tanks and aerosols than in the refilling dispenser [9]. In their latest investigations, the levels of harmful metals in aerosols were found to be largely dependent on the device power and type (open or closed system), and increased with higher power and the use of an open-system device [10]. 

Despite the results obtained in the metal emission studies, the risk assessments performed to evaluate the harmful potential from exposure to metals found in aerosol samples did not reveal any significant adverse health effects for smokers switching to ECs and other EC users with a typical EC usage pattern [11,12]. Therefore, further research is still required to evaluate the relationship between EC usage pattern and biomarkers and to compare metal biomarker levels in EC users, non-users and smokers. Smoking is seen as a significant source of metal exposure, however, in most cases only Cd and Pb were found in higher levels in smokers’ urine and blood compared to non-smokers. The increase of other elements was questioned and even an inverse relationship was observed.

There are very few studies regarding the assessment of such an exposure using specific biomarkers in EC-user population. Recently, Aherrera et al. [13] have found that Ni and Cr, which are components of e-cigarette heating coils, may transfer to aerosol and increase metal internal dose, indicating a positive association with the corresponding Ni and Cr level in urine, saliva and exhaled breath condensate. In another study, cadmium level in the blood of EC users was found to be significantly lower than in cigarette smokers but doubtful results were found for blood lead [14]. Badea et al. [15] found that EC users presented the highest concentrations of selenium, silver, and vanadium in blood serum, since beryllium, europium and lanthanides were detected more frequently among e-cigarette users than in cigarette smokers. Due to very few metal biomarker studies, our aim was to assess whether the e-cigarette use increases the metal concentration in urine in comparison to non-smokers and smokers of conventional cigarettes. In our studies, levels of 12 metals found in literature to be present in aerosol produced during the use of ECs were investigated. It was also checked whether there was any positive association between the EC usage pattern and the metal level in urine among EC-only users. 

## 2. Methods

For the conducted study, 90 volunteers (52 men and 38 women) at the age of 19 and 39 years were enrolled. The volunteers were divided into four groups: Smokers who smoked cigarettes for at least 2 years, dual users who smoked conventional cigarettes for at least 2 years and used ECs for at least 6 months, EC users who used ECs for at least 6 months and were former smokers with minimum duration of smoking cessation of 6 months and who directly switched from combustible cigarettes to ECs after smoking for at least 2 years, and non-smokers. The smoking status was verified by measuring exhaled CO in the morning hours before the examination. The questionnaire involved questions regarding the amount of smoking and/or amount of e-liquids used a day, type of liquids (nicotine content and flavoring) and the used EC device. Finally, due to the lack of some data, 88 participants were selected for taking part in the study. The characteristics of the studied population is presented in Table 1. Almost all participants lived in Sosnowiec (city of 240,000 inhabitants) and the vicinity located in southern Poland within the Silesia-Basin Metropolis. They declared that they were not occupationally exposed to metals, and their living area was not close to significant point emission sources of heavy metals. It was assumed that environmental exposure to investigated elements was similar across the studied population. The study design was approved by the Bioethics Committee of the Institute of Occupational Medicine and Environmental Health in Sosnowiec.

### 2.1. ECs Used by Participants

Participants from the e-cigarette group used 12 different brands of 2nd-generation (23 individuals) and 3rd-generation (2 individuals) ECs. The 3rd generation devices give users more flexibility, allowing them to set different output voltages and control temperature of the coil, depending on the chipset used in the device. These devices deliver higher output voltage, allowing in some cases to use the device with wattage higher than 200 W. The nicotine concentrations of the applied e-liquid were as follows: 0.1–0.4% (1 individual); 0.6–0.9% (8 individuals); 1.0–1.5% (1 individual); 1.6–2.4% (10 individuals); >2.4% (4 individuals). Among the 6 groups of flavorings, most of the individuals used fruit taste (10 individuals), followed by tobacco taste (6 individuals), tea/coffee taste (5 individuals) and menthol taste (3 individuals). Participants from the dual-user group used 8 different brands of 2nd-generation (12 individuals) and 3rd-generation (1 individual) ECs. The nicotine concentrations of e-liquid were as follows: 0.1–0.4% (1 individual); 1.6–2.4% (8 individuals); >2.4% (4 individuals). From among 7 groups of flavorings, most of the individuals used menthol taste (5 individuals), which was followed by fruit taste (4 individuals), tea/coffee taste (3 individuals) and tobacco taste (1 individual).

### 2.2. Determination of Metal Levels in Urine

Morning urine samples were used to determine metal concentrations in volunteers. A total of 5 mL of urine was mineralized with 2.5 mL of concentrated nitric acid and diluted finally to 25 mL. The determination of the 11 elements in the collected samples was performed using an ICP-MS method (Perkin Elmer Elan 6100 DRC-e, Shelton, USA) with standard equipment: Quartz torch, nickel cones and cross-flow nebulizer. Optimization of the spectrometer was carried out daily with a 10 µg/L solution (Mg, Cu, Rh, Cd, In, Ba, Ce, Pb, U) in 1% nitric acid Elan 6100 Setup/Stab./Masscal. solution from Perkin-Elmer. The spectrometer operating parameters were as follows: RF power 1125 W, plasma gas flow 15 L/min, nebulizer gas flow 0.77–0.82 L/min, auxiliary gas flow 1.15 L/min, nebulizer cross-flow, sample flow 1 mL/min, scanning mode peak hopping, dwell time 100 ms, sweeps/reading 20, number of replicates 3. Element concentrations were determined using the internal standard method based on ^103^Rh, and in the case of vanadium the following correction equation was used: −3.127 (^53^Cr-0.1134 ^52^Cr). The limit of detection (LOD) for particular metals were ^51^V 0.5 µg/L, ^55^Mn 0.30 µg/L, ^59^Co 0.35 µg/L, ^60^Ni 0.55 µg/L, ^107^Ag 0.010 µg/L, ^114^Cd 0.05 µg/L, ^138^Ba 0.5 µg/L, ^115^In 0.005 µg/L, ^208^Pb 0.45 µg/L, ^121^Sb 0.05 µg/L. Cr levels in the urine samples were determined by atomic absorption spectrometry using electrothermal atomization (ETAAS) and Zeeman-type background correction (PerkinElmer 4100ZL, Bodenseewerk Perkin-Elmer, Ueberlingen, Germany) after 1:1 dilution of urine with 5% nitric acid. LOD for Cr was 0.06 µg/L. The applied method validation details are presented in the Supplementary Materials. Concentration of metals in urine was expressed directly per volume basis as well as in relation to creatinine concentration, which was determined by Jaffé reaction method. Both units were used because reference values for pollutants in urine are usually expressed as μg/L urine. Multiple blank samples were included in each analysis series to check and control the possible contamination.

### 2.3. Statistical and Mathematical Calculation

The calculations were performed using STATISTICA 13 (TIBCO Software Inc., Palo Alto, CA, USA). As appropriate, the geometric mean with 5th–95th percentiles and median with interquartile range were chosen for the presentation of characteristics and measures distributed across specified groups of data. The initial between-group differences in the means and proportions were tested by the Kruskal–Wallis rank test and F-test, respectively. Spearman rank correlation coefficient was used to assess the correlation between specified independent variables and the urine element level. Analysis of covariance was used to compare the adjusted values of the elements in urine across the specified groups and the association between the EC usage pattern and biomarkers was evaluated using multiple linear regression. Due to the non-normal distribution of data, the values were natural log transformed to the approximate a normal distribution. The analysis was controlled by age, sex, and BMI as a priori covariance (Model 1) and additionally by the amount of nicotine liquids used per week and the number of cigarettes per day (Model 2). The amount of liquid per week seems to be the most reliable and simple parameter to assess EC usage intensity in the applied questionnaire and finally it was chosen to build Model 2. EC usage pattern parameters, such as the number of puffs for a single puffing session, number of e-cigarette puffs per day and the amount of nicotine liquid per week were used as independent variables in the multiple linear regression analysis. The results were considered significant at *p* < 0.05. 

## 3. Results

A description of the study population (geometric mean and 5th-95th percentile) and summaries of the urine analysis results (median and interquartile range) are presented in Table 1 and Table 2, respectively. All the sample levels under the method detection limit were replaced by the limit of detection divided by square root of two. The initial non-parametric statistics revealed that the levels of determined elements were not significantly different among the specified groups. There was also no difference in the levels of biomarkers between EC users and dual users. After controlling for age, BMI and sex, a significantly higher level was observed only for urinary Sb in the dual-user group compared to non-smokers (Table 3). In EC group the Spearman correlation coefficient between the element concentration in urine and the EC usage pattern was statistically significant only for Ba μg/creat vs. number of puffs (0.535), Sb μg/L vs. amount of liquid per week (0.664) and with some tendency (p ≤ 0.1) for Ni μg/creat vs. number of puffs and for Mn μg/creat vs. e-cigarettes per day. The multiple regression analysis revealed that only the number of puffs was positively associated with Ba μg/creat (*β* = 0.691, *p* = 0.001) and Ni/creat (*β* = 0.453, *p* = 0.032).

## 4. Discussion 

The aim of the study was to assess whether the use of ECs increases the concentration of some elements excreted in urine. With a few exceptions, urine is usually the main route of metal elimination and the increased exposure gives rise to higher metal concentration in urine [16]. ECs were recognized as a possible source of metal exposure since their relatively high concentrations were reported in both nicotine liquids and aerosols, but not in liquids from dispensers for some types of devices. The number of metals appeared to be large and even exceeding the combustible tobacco cigarettes [4]. This can be the result of a direct contact of the liquid with several parts of the EC device components (joints, wires, complex alloy, and plastics). Taking into account the exposure of EC users to the analyzed metals, there is a need to consider their levels in aerosol which is inhaled and enters the body. However, their internal dose depends on many parameters and characteristics linked to vaping. In our study, element concentration in urine was used as a biomarker of exposure to some elements (Ba, V, In, Ag, Mn, Co, Ni, Cr, Sb, Cd, and Pb) encountered in EC aerosols in order to find the possible contribution to their internal dose. Our results indicated that there were no significant differences in the urine concentration of the studied elements among EC users, non-smokers and smokers.

Exposure to barium is common since its abundance in the earth’s crust is estimated at 0.04–0.05%. Our results for median urinary level of barium is nearly twice as high as in the National Health and Nutrition Examination Survey (NHANES) program where medians of 1.21 μg/L and 1.32 μg/g creatinine were reported for all populations [17]. In other studies, higher levels of urinary barium in the general population were also reported, similar to our results [18]. At high-exposure barium is responsible for renal intoxication, hypertension, cardiac malfunction and pneumoconiosis but its toxicity at a low exposure is unknown. No differences among all the studied groups were found, except for somewhat higher levels of urinary barium observed in the dual group. Since barium is present in relatively high concentrations in the environment, it is possible that e-cigarette liquids and devices are simply contaminated by this metal with low contribution to internal dose compared to other sources of exposure (air, food and drinking water). However, barium in urine was found to be positively correlated with the number of puffs in EC-only users, indicating a possible association. This association does not necessarily indicate that e-cigarettes are an important source of barium exposure but it may simply be a marker of some other source/pathway of exposure, for example, frequent hand-to-mouth contact. Direct exposure from aerosol is also a possible explanation according to the recent study in which barium was present in aerosol samples in the amount of 0.001–0.010 μg/10 puffs [4].

Vanadium is another metal which was detected in a similar amount in aerosol samples as barium. It is a ubiquitous element, which is present in soil, water and the atmosphere. Excessive exposure can cause adverse effects on physiology and morphology of tissues, including male reproductive toxicity [19]. The reference range reported by Guidotti et al. 1997 and used by the Trace Element and Environmental Toxicology Laboratory at the University of Alberta Hospitals in Canada was 0–9 μg/L in 24 h collection urine samples, which is within the results obtained in our study in spot urine samples [20]. However, some recent works concerning metals and metalloids in the general population reported much lower results [19,21,22]. This may be due to regional variability in the extent and exposure sources. Exposure to vanadium is predominantly via food. In the air the predominant source of vanadium is fossil fuel combustion, especially crude oil. Since the participants originated from the urban area with a long history of heavy industry and mining activity, higher background exposure compared to other regions is possible.

Antimony is a well-known toxic substance at high doses, with a possibility to be carcinogenic to humans (group 2B IARC). Its toxicological properties are similar to arsenic. As it has been found, antimony in urine may increase the prevalence of peripheral artery disease at environmental background levels [23]. It may enter the environment from natural sources and from its use in industry (fire-retardant in textiles and plastics, brake pads). Little is known about internal exposure of the general population. In German children, the median for urine antimony was 0.11 μg/L [24]. In our study, lower levels of Sb were found in all groups, which was similar to other studies concerning the general population [17,21] but a significant increase was observed in dual users in Model 1. In the Spearman rank correlation, a positive association was found between Sb in urine and the amount of nicotine liquid used per week in EC-user group, which may indicate the possibility of additional exposure to Sb from ECs.

Chromium and nickel may be typical components of heating coil in e-cigarette devices. They were found in liquids and aerosols in wide ranges of concentration [25,26,27]. Chromium is an important metal involved in glucose metabolism and relatively high levels of the most prevalent Cr (III) compounds are needed to cause the toxic effect. Cr (VI) species, which are the most toxic due to carcinogenic properties, are not very likely to occur in the EC aerosol because of their low stability. It is commonly known that an allergic reaction to nickel is the most common hypersensitivity affecting 10–20% of the population. Various nickel compounds are classified as carcinogenic to humans. Conventional tobacco contains a relatively high level of nickel but after burning it remains mainly in ash. Aherrera et al. [13] found that higher urine nickel levels in EC users were linked to shorter time to the first vape from waking, more frequent change of coils, higher urinary cotinine and higher Ni levels in aerosol samples from their personal device. In the same study, urine chromium was not significantly associated with any investigated EC usage pattern. In our study, no significant differences in nickel and chromium excretion in urine among EC users compared to non-smokers and smokers were observed. However, nickel in urine was associated with the number of puffs in EC-only users after controlling for age, BMI and sex. Nevertheless, the effect for nickel might be overshadowed by environmental exposure since nickel was found in urine at a relatively higher level compared to other studies [21,22,28]. The effect of smoking habits on urine nickel levels is questioned and in many studies it was independent of this factor compared to our study. Chromium in urine was found in the low range of the existing concentrations for this metal, indicating a low possibility of substantial exposure to chromium from e-cigarette use [21,28].

Manganese is a neurotoxic metal but acute and intermediate exposure to Mn may also affect the respiratory system. Its elimination is predominantly via bile and only approximately 0.01% of Mn is eliminated via urine. Average urinary excretion levels are believed to be the most indicative of recent exposure, although with a high degree of variability. However, urine levels are not a reliable predictor of exposure to manganese on an individual basis [29]. In the general population the concentration of manganese in urine is usually <1 μg/L or <1 μg/g creatinine, which is within the results obtained in our study [21,22]. In no group was the average manganese level in urine significantly different, and in EC-only users urinary manganese was not correlated with any of the EC usage patterns.

Ag is another metal the excretion of which is primarily biliary. Nearly all the results for the tested urine samples were below the detection limit, except for very few cases. Indium, in turn, was found to be at very low levels in urine samples (<0.02 μg/L) and no differences were observed among the studied groups.

Cobalt is a rare but widely distributed metal in nature. Although Co plays an important role as a metal containing vitamin B_12_, chronic excessive exposure is reported to induce an immune-mediated response and local adverse health reactions like pulmonary syndrome and thyroid abnormalities [30]. Co concentration in urine did not vary across the studied groups with the concentration median <1 μg/L and <1 μg/g creatinine—the level which is typical for the general population [21].

Nephrotoxicity and carcinogenicity are the main toxic effects associated with the exposure to cadmium. Cadmium is a metal, which despite a low content in tobacco, is characterized by a high rate of transfer from tobacco to cigarette smoke [31]. In many studies, cadmium in urine is higher in smokers, being the most significant source of increased cadmium body burden [21]. Cadmium accumulates in kidneys and due to very long half-life, urinary cadmium is a biomarker of long-term exposure. Surprisingly, in our study, no significant differences were found across the studied groups with regard to the concentration of cadmium in urine. The main reason for this may be different smoking duration, living in urbanized and industrial areas and the relatively young age of the individuals. The medians in all studied groups were low and similar to those obtained in other studies for the general population. No significant effect of smoking on urinary cadmium was found in the adult Flemish population study, either, which was explained by a large overlap in the number of cigarettes smoked per week [32]. Similarly, cadmium in urine concentrations in Czech adults were not influenced by smoking habits [33]. In contrast to the urine cadmium level, blood cadmium indicates recent exposure and our previous study showed a rapid decrease in blood cadmium level in EC users compared to smokers and duals [14]. Urinary lead, in turn, reflects recent exposure with a high degree of variability. Due to the lower environmental exposure, tobacco contribution to the exposure to lead is much clearer. In studies of the general population, lead in urine was significantly higher in smokers than in non-smokers [21]. Marginally significant higher level in smokers was found in the first Model 1 (*p* = 0.07) but the level in EC users and duals was not significantly different compared to non-smokers. Similar results were obtained in the recent study using blood lead as a biomarker [14]. Lead is a metal of special concern due to its toxicity and reports on high levels in nicotine liquids and aerosols indicating the possibility of significant exposure level [8,9]. Our results may indicate that EC users are less exposed to lead than smokers of conventional cigarettes.

Olmedo et al. [9] suggest that using e-cigarettes instead of conventional cigarettes may result in lower exposure to Cd but not to other hazardous metals found in tobacco. Based on this study, Farsalinos and Rodu calculated that the amount of liquid needed to exceed the safety limit is unrealistically high for the studied metals compared to the real use of this product [12]. As mentioned earlier, the first risk assessment analysis regarding metals emitted from ECs indicated that the EC use was not expected to pose a significant health risk to smokers switching to ECs [11]. Our study confirms this view and shows that the exposure to metals, which was assessed by their concentrations in urine, is not significantly higher in EC users compared to smokers and even non-smokers. E-vapor aerosols generated under machine puffing conditions for determination of toxicants by various analytical chemistry techniques or in vitro/in vivo toxicological assays are not generally representative for aerosols generated under human vaping behaviors. Moreover, they do not account for human absorption, distribution, metabolism, and excretion. The procedure described in this article accounts for all these processes. Moreover, it includes the effects of human puffing behaviour. Thus, the results obtained using our procedure give a complete picture of the intake of metals during vaping. In the recently published study, liquids of different brands and flavors have been analyzed, and it has been confirmed that their contact with the device components caused metal contamination of the liquid and the risk of potential exposure [34]. However, it is difficult to predict the exposure to metals on the basis of the metal concentration in the liquid due to different transfer efficiency from the liquid to aerosol, which depends on many variables. The authors concluded that in order to calculate the ranges of the potential exposure, a high number of replicates of liquids and devices should be analyzed, taking into account different vaping conditions. Instead, in our study a biomonitoring technique was used, the results of which reflect the real exposure and enable the estimation of the potential of the inhaled aerosols to cause adverse health effects.

The presented study has several limitations. Firstly, the studied group was relatively small, especially in the case of duals (due to the difficulties in finding participants using both products), so there is a possibility that the analysis was underpowered to detect significant differences in the concentrations of the analyzed elements. Due to the small sample size and many different brands (and devices) used by the EC users, the results may not be representative for the overall population and should be interpreted with caution. Extending our study to a much larger sample size will be necessary to confirm the results and associations between the various factors investigated and the metal levels in urine. Secondly, the participants live in a densely populated and highly urbanized area with a long history of heavy industry and mining activity. Because of this, the background exposure to metals is probably higher than in other regions and may modify the effect. Moreover, what was assumed in the carried-out study was low variability in diet, which might influence the exposure to metals considerably since in the general population it was mainly via food. The urine concentration reflects the exposure from all sources, including inhalation pathway which is, in turn, essential for EC users to assess toxicological effects. Additional limitation is relatively short cigarette smoking time and EC usage duration in individuals participating in our study, and another one is CO measurement as a biomarker of smoking as it reflects only a short-term effect of smoking and does not indicate whether individuals have been using cigarettes in the earlier period or not. Finally, it should be noted that in the statistical analysis, the data obtained from the questionnaire were used, thus the quality of the responses might have had an influence on the obtained results. 

## 5. Conclusions

In conclusion, the results obtained in our study indicate that concentrations of elements, which were determined in EC users’ urine, were not significantly different compared to concentrations in non-smokers. This finding shows that use of a wide variety of e-vapor products does not lead to the increased uptake of metals by vapers who use such products. 

## Figures and Tables

**Table 1:** Participant characteristics.

Characteristic	Non-Smokers (*n* = 25)	EC Users (*n* = 25)	Dual EC Users- Cigarette Smokers (*n* = 13)	Cigarette-only Smokers (*n* = 25)	*p*-Value
Male/Female ( *n*)	17/10	13/12	4/9	14/11	0.30
Age (years)	28.9 (21–39)	28.8 (20–39)	26.2 (18–35)	28.1 (21–39)	0.64
BMI (kg/m^2^)	22.2 (19.0–25.2)	23.8 (19.3–31.0)	24.3 (19.5–34.3)	23.7 (19.4–28.9)	0.11
Cigarettes per day, (*n*)	NA	NA	6.2 (1–15)	13.6 (10–20)	0.002
Number of e-cig. puffs per day (*n*)	NA	33.1 (10–99)	27.2 (5–120)	NA	0.67
Number of puffs per single session (*n*)	NA	6.2 (2–20)	6.5 (2–15)	NA	0.91
Amount of liquid (mL/week)	NA	15.9 (8–35)	11.6 (5–20)	NA	0.12

Note: Geometric Mean (5th–95th percentile), NA—not applicable

**Table 2:** Median (interquartile range) of urinary element concentration in particular groups.

**Element** **(μg/L)**	**Group**				***p*-Value**
	**Non-Smokers**	**EC-Users**	**Duals**	**Smokers**	
Cr	0.19 (0.10–0.31)	0.11 (0.07–0.19)	0.19 (0.07–0.29)	0.12 (0.06–0.29)	0.35
V	9.1 (5.5–13.7)	12.1 (7.6–14.72)	10.6 (8.3–14.4)	13.0 (8.1–19.1)	0.33
Ba	2.4 (1.6–5.0)	3.2 (1.6–7.5)	5.6 (3.1–7.5)	3.5 (1.9–5.0)	0.25
In	0.005 (0.003–0.010)	0.005 (0.003–0.015)	0.01 (0.003–0.015)	0.005 (0.003–0.025)	0.26
Ag	<LOD (<LOD–<LOD)	<LOD (<LOD–<LOD)	<LOD (<LOD–<LOD)	<LOD (<LOD–<LOD)	-
Co	0.63 (0.37–1.51)	0.82 (0.39–1.31)	0.74 (0.48–1.23)	0.84 (0.49–1.21)	0.97
Ni	5.1 (3.0–8.6)	6.3 (3.4–12.4)	7.2 (3.6–10.0)	6.5 (5.0–9.9)	0.79
Mn	1.27 (0.81–1.62)	1.25 (0.97–1.78)	0.81 (0.76–1.93)	1.24 (0.72–1.53)	0.83
Pb	1.16 (<LOD–1.87)	1.38 (<LOD–2.05)	1.23 (<LOD–1.40)	1.77 (1.01–2.34)	0.28
Sb	0.05 (<LOD–0.08)	0.07 (<LOD–0.08)	0.08 (0.06–0.27)	0.05 (<LOD–0.08)	0.26
Cd	0.37 (0.29–0.53)	0.44 (0.29–0.64)	0.42 (0.21–0.68)	0.43 (0.18–0.63)	0.89
**Element** **(μg/g Creatinine)**	**Group**				***p*-Value**
	**Non-Smokers**	**EC-Users**	**Duals**	**Smokers**	
Cr	0.14 (0.08–0.21)	0.06 (0.05–0.11)	0.12 (0.06–0.34)	0.09 (0.05–0.24)	0.16
V	7.0 (5.8–10.5)	6.9 (5.5–8.2)	7.8 (6.4–8.6)	8.5 (5.7–10.9)	0.58
Ba	2.3 (1.1–3.2)	2.01 (1.2–4.4)	3.66 (2.04–4.45)	2.19 (1.24–3.06)	0.35
In	0.004 (0.002–0.011)	0.005 (0.002–0.008)	0.006 (0.003–0.008)	0.004 (0.002–0.016)	0.41
Ag	<LOD (<LOD–<LOD)	<LOD (<LOD–<LOD)	<LOD (<LOD–<LOD)	<LOD (<LOD–<LOD)	-
Co	0.51 (0.34–0.89)	0.46 (0.30–1.05)	0.44 (0.35–0.58)	0.61 (0.31–0.71)	0.88
Ni	5.02 (3.1–7.2)	5.23 (2.41–6.72)	5.24 (3.03–6.55)	4.24 (2.74–6.98)	0.98
Mn	0.84 (0.59–1.60)	0.80 (0.60–1.15)	0.61 (0.51–1.43)	0.71 (0.49–0.92)	0.56
Pb	0.68 (<LOD–1.03)	0.66 (<LOD–1.14)	0.3 (<LOD–0.91)	0.98 (0.63–1.48)	0.31
Sb	0.02 (<LOD–0.05)	0.04 (<LOD–0.05)	0.04 (0.03–0.13)	0.03 (<LOD–0.06)	0.41
Cd	0.35 (0.20–0.42)	0.29 (0.20–0.41)	0.26 (0.19–0.45)	0.28 (0.20–0.51)	0.98

LOD—limit of detection.

**Table 3:** Ratio of geometric means (95% confidence interval-CI) in Models 1 and 2 with non-smokers as a reference group *.

Element/Group	Geometric Mean Ratio (95% CI) μg/L		Geometric Mean Ratio (95% CI)	
			μg/g Creatinine	
	Model 1	Model 2	Model 1	Model 2
Cr				
Non-smokers	1 (ref)	1 (ref)	1 (ref)	1 (ref)
EC users	0.78 (0.52, 1.12)	1.06 (0.51, 2.20)	0.77 (0.54-1.10)	1.20 (0.59, 2.43)
Duals	1.26 (0.79, 2.02)	1.16 (0.67, 2.03)	1.33 (0.84, 2.11)	1.20 (0.70, 2.06)
Smokers	0.98 (0.68, 1.43)	0.65 (0.29, 1.50)	0.92 (0.64, 1.32)	0.52 (0.23, 1.16)
*p*-value	0.55	0.57	0.41	0.27
V				
Non-smokers	1 (ref)	1 (ref)	1 (ref)	1 (ref)
EC users	1.01 (0.82, 1.24)	0.93 (0.61-1.41)	0.98 (0.85, 1.14)	1.02 (0.77, 1.39)
Duals	0.98 (0.75, 1.28)	1.00 (0.73-1.38)	1.01 (0.84, 1.22)	1.03 (0.81, 1.27)
Smokers	1.13 (0.91, 1.39)	1.27 (0.79, 2.04)	1.05 (0.90, 1.22)	1.02 (0.73, 1.42)
*p*-value	0.6	0.71	0.88	0.96
Ba				
Non-smokers	1 (ref)	1 (ref)	1 (ref)	1 (ref)
EC users	0.95 (0.68, 1.34)	1.04 (0.53, 2.05)	0.93 (0.69, 1.25)	1.16 (0.64, 2.09)
Duals	1.32 (0.86, 2.05)	1.32 (0.79, 2.22)	1.37 (0.93, 2.00)	1.34 (0.85, 2.11)
Smokers	0.95 (0.67, 1.34)	0.90 (0.42, 1.94)	0.88 (0.65, 1,20)	0.72 (0.37, 1.42)
*p*-value	0.59	0.74	0.46	0.46
In				
Non-smokers	1 (ref)	1 (ref)	1 (ref)	1 (ref)
EC users	0.94 (0.59, 1.51)	2.21 (0.88, 5.53)	0.92 (0.55, 1.52)	2.46 (0.93, 6.54)
Duals	0.98 (0.54, 1.80)	0.84 (0.42, 1.69)	1.01 (0.53, 1.94)	0.85 (0.40, 1.79)
Smokers	1.12 (0.69, 1.81)	0.40 (0.14, 1.13)	1.04 (0.63, 1.75)	0.32 (0.11, 0.97)
*p*-value	0.97	0.32	0.99	0.04
Co				
Non-smokers	1 (ref)	1 (ref)	1 (ref)	1 (ref)
EC users	1.02 (0.78, 1.33)	1.20 (0.70, 2.04)	1.01 (0.81, 1.25)	1.35 (0.88, 2.06)
Duals	1.07 (0.76, 1.51)	1.04 (0.69, 1.56)	1.12 (0.85, 1.48)	1.07 (0.77, 1.48)
Smokers	0.98 (0.75, 1.29)	0.84 (0.46, 1.54)	0.93 (0.74, 1.16)	0.68 (0.42, 1.10)
*p*-value	0.96	0.91	0.83	0.42
Ni				
Non-smokers	1 (ref)	1 (ref)	1 (ref)	1 (ref)
EC users	1.02 (0.76, 1.36)	1.33 (0.75, 2.35)	0.99 (0.77, 1.28)	1.48 (0.89, 2.44)
Duals	1.03 (0.71, 1.48)	1.00 (0.64, 1.54)	1.05 (0.76, 1.47)	1.01 (0.69, 1.48)
Smokers	1.05 (0.79, 1.42)	0.81 (0.42, 1.55)	0.99 (0.76, 1.28)	0.65 (0.37, 1.15)
*p*-value	0.92	0.79	0.99	0.44
Mn				
Non-smokers	1 (ref)	1 (ref)	1 (ref)	1 (ref)
EC users	1.10 (0.91, 1.34)	1.06 (0.73, 1.57)	1.09 (0.87, 1.37)	1.21 (0.76, 1.91)
Duals	0.98 (0.76, 1.26)	0.94 (0.70, 1.26)	1.03 (0.77, 1.39)	0.98 (0.69, 1.38)
Smokers	0.93 (0.76, 1.14)	0.91 (0.59, 1.41)	0.88 (0.70, 1.12)	0.74 (0.44, 1.25)
*p*-value	0.75	0.95	0.71	0.72
Pb				
Non-smokers	1 (ref)	1 (ref)	1 (ref)	1 (ref)
EC users	1.01 (0.71, 1.45)	0.92 (0.45, 1.89)	0.97 (0.69, 1.37)	0.99 (0.49, 1.97)
Duals	0.76 (0.48, 1.21)	0.65 (0.38, 1.12)	0.78 (0.51, 1.21)	0.64 (0.38, 1.07)
Smokers	1.40 (0.97, 2.01)	1.31 (0.58, 2.95)	1.31 (0.93, 1.83)	1.06 (0.50, 2.23)
*p*-value	0.31	0.41	0.43	0.37
Sb				
Non-smokers	1 (ref)	1 (ref)	1 (ref)	1 (ref)
EC users	0.96 (0.73, 1.27)	0.90 (0.52, 1.57)	0.95 (0.68, 1.33)	1.07 (0.54, 2.13)
Duals	1.67 (1.16, 2.40)	1.46 (0.96, 2.24)	1.74 (1.13, 2.68)	1.46 (0.87, 2.45)
Smokers	0.87 (0.65, 1.15)	0.83 (0.44, 1.57)	0.82 (0.58, 1.16)	0.64 (0.30, 1.37)
*p*-value	0.03	0.26	0.07	0.24
Cd				
Non-smokers	1 (ref)	1 (ref)	1 (ref)	1 (ref)
EC users	1.06 (0.84, 1.34)	0.96 (0.60, 1.54)	1.04 (0.87, 1.24)	1.08 (0.76, 1.54)
Duals	1.06 (0.78, 1.44)	1.12 (0.78, 1.60)	1.11 (0.89, 1.40)	1.15 (0.88, 1.51)
Smokers	1.02 (0.80, 1.29)	1.21 (0.71, 2.06)	0.96 (0.80, 1.15)	0.98 (0.65, 1.46)
*p*-value	0.76	0.69	0.61	0.61

* Model 1 adjusted for age, BMI and sex; Model 2 additionally adjusted for the amount of nicotine liquid per week and the number of cigarettes per day. *p*-value for effect.

## Supplementary Materials

The following are available online at https://www.mdpi.com/1660-4601/17/6/1877/s1, Table S1: Results of repeatability and reproducibility studies. Table S2: Recovery experiment from urine sample. Table S3: Analytical Results for the Certified Reference Material NIST 1643e. Table S4: Analytical characteristic data of the Cr determination method.
