# Supplementary material for: Metal Concentration Assessment in the Urine of Cigarette Smokers Who Switched to Electronic Cigarettes: A Pilot Study
Source: Int J Environ Res Public Health. 2020 Mar 13;17(6):1877. doi: 10.3390/ijerph17061877 (PMC7143212; doi:10.3390/ijerph17061877)
Supplement: Supplementary file 1 [file ijerph-17-01877-s001.pdf]

Supplementary Materials

The optimal condition for multi-elemental analysis were establish based on results on standard deviation of obtained results, spectral and matrix interferences and measurement time of each element.

Radio frequency (RF) power has been determined for highest efficiency of ion (M^+) formation as well as to limit MO^+ ion formation. Too high plasma power results in increased formation of double-charged ions (M^{++}). Auxiliary gas flow value is set according to the manufacturer's instructions.

According to ISO 17,294 sample flow is set to the lowest possible to limit the matrix effects.

The efficiency of the device was checked in each case in relation to sensitivity, precision, background concentration of oxides and ions with double charge. The instrument performance is also checked daily before measuring samples. This procedure involves measuring an optimization solution (Elan 6100 Setup/Stab./Masscal. Solution) with a concentration 10 μg/L of appropriate elements (Mg, Cu, Rh, Cd, In, Ba, Ce, Pb, U) in 1% HNO3. The spectrometer was optimized to provide maximal intensity for ^{24}Mg, ^{115}In, ^{238}U, and minimal values for CeO/Ce (below 3%) and Ba^{2+}/Ba (below 3%). According to manufacturer guidelines this procedure is necessary to control and improve parameters of ICP-MS apparatus (for example nebulizer gas flow value).

Limits of detection of each analytes was determined as three time value of standard deviation for the blank sample, which was acidified water sample, used to prepare calibration solution and dilution of real samples. The limits of quantification was expressed as three times value of limit of detection.

Validation

All standards and blank were prepared daily by using analytical balance, directly before sample analysis. In order to minimalize matrix effects, standards, blanks and samples were measured using Rh as internal standard (10 μg/L, Merck, Germany). Solution of 10 μg/L Rh was move into all solutions on line, by second tubing on peristaltic pomp.

Calibration linearity was checked with a standard solution different than that used for calibration. During the validation of the method repeatability and reproducibility of the method was studied. The following Table S1 presents the results.

Table S1. Results of repeatability and reproducibility studies.

Element	*n*	O	m [μg/L]	S_R [μg/L]	V_R[%]	S_r [μg/L]	V_r [%]
V	59	0	9.915	0.376	3.8	0.317	3.2
Mn	120	2	10.365	1.077	10.4	0.655	6.3
Co	116	1	10.204	0.470	4.6	0.468	4.6
Ni	120	0	10.271	0.477	4.6	0.402	3.9
Ag	61	0	9.977	0.690	6.9	0.319	3.2
Cd	115	1	10.357	0.554	5.3	1.438	13.9
Ba	109	1	10.494	0.861	8.2	0.679	6.5
Pb	87	5	10.338	1.436	13.9	1.167	11.3
Sb	88	3	10.326	0.659	6.5	0.695	6.9

n—number of results; O—number of results rejected after Grubbs test for coarse error, 95% confidence level; m—average concentration obtained for measuring n samples B; S_R—standard deviation of reproducibility; V_R—relative standard deviation of reproducibility; S_r—standard deviation of repeatability; V_r—relative standard deviation of repeatability.

To verify recovery of the measured elements the real samples were enforced by known amount of standards and analyzed according to procedure. The results were showed in the Table S2 below:

Table S2. Recovery experiment from urine sample.

Element	Recovery [%]
V	101
Ba	116
In	118
Ag	103
Co	105
Ni	96
Mn	104
Pb	84
Sb	105
Cd	98

The 1643e the National Institute of Standards and Technology (NIST) reference material was used to verify the quality of the analysis. Certified values of concentration from this reference material are available for almost all analyzed elements in this work. Recovery experiments of Certified Reference Material (CRM) NIST 1643e are presented in the following Table S3. The determined values agreed well with certified values.

Table S3. Analytical Results for the Certified Reference Material NIST 1643e.

Element	NIST 1643-e		*n*	Measured Concentration	Recovery
	Concentration [μg/L]	Uncertainty [μg/L]		[μg/L]	[%]
Mn	38.97	0.45	12	42.12	108
Ni	62.41	0.69	12	66.75	107
Co	27.06	0.32	12	29.67	109
Cd	6.568	0.073	12	6.36	97
V	37.86	0.59	12	42.08	111
Ag	1.062	0.075	12	1.01	95
Pb	19.63	0.21	12	20.74	106
Ba	544.2	5.8	12	561.89	103
Sb	58.30	0.61	12	57.8	99

Chromium in urine was determined by means graphite furnace atomic absorption spectrometry methods (GFAAS) using PerkinElmer 4100ZL atomic absorption spectrometer equipped with transversely-heated graphite atomizer with integrated platforms, a longitudinal Zeeman background correction system and furnace autosampler. For analysis optimized time-temperature program has been established with pyrolysis and atomization temperature 1500 and 2400 °C, respectively. Determination of chromium was performed after equal (1:1) dilution of urine in matrix modifier solution containing 0.15% m/v Mg $(NO_3)_2$ in 5% v/v nitric acid and calibration against matrix-match calibration standards. The peak height was used as a analysis mode with smoothing the signal. Limit of detection was determined as three time value of standard deviation for the blank sample, which was matrix modifier solution diluted with deionized water. Additionally, repeatability and recovery was studied using control material and enforced samples. The results present the following Table S4:

Table S4. Analytical characteristic data of the Cr determination method.

Parameter	Value
Detection limit	0.06 μg/L
Recovery (*n* = 10)	113%
RSD (*n* = 10)	5.5%
